# Supplementary material for: Ultrafast light-induced dynamics in the microsolvated biomolecular indole chromophore with water
Source: Nat Commun. 2022 Dec 3;13:7462. doi: 10.1038/s41467-022-33901-w (PMC9718776; doi:10.1038/s41467-022-33901-w)
Supplement: Supplementary file 1 — Supplementary Information [file 41467_2022_33901_MOESM1_ESM.pdf]

Supplementary Information: Ultrafast light-induced dynamics in the microsolvated biomolecular indole chromophore with water

Jolijn Onvlee,[1,2] Sebastian Trippel,[1,2] and Jochen Küpper[1,2,3]

[1] *Center for Free-Electron Laser Science, Deutsches Elektronen-Synchrotron DESY, Notkestraße 85, 22607 Hamburg, Germany*
[2] *Center for Ultrafast Imaging, Universität Hamburg, Luruper Chaussee 149, 22761 Hamburg, Germany*
[3] *Department of Physics, Universität Hamburg, Luruper Chaussee 149, 22761 Hamburg, Germany*

(Dated: 2022-09-08)

Email: jochen.kuepper@cfel.de; website: `https://www.controlled-molecule-imaging.org`

Current address of J. O.: Institute for Molecules and Materials, Radboud University, Nijmegen, The Netherlands

SUPPLEMENTARY NOTE 1: SPECIES SEPARATION

Using the electrostatic deflector, we spatially separated indole(H_2O) from the other species present in the molecular beam [1–4], as shown in Supplementary Figure 1 a. The orange line shows the molecular beam profile with the deflector switched off. In this case, the profiles are practically identical for all molecular species in the beam. However, when the deflector is switched on by applying high voltage to the electrodes, the molecules experience the resulting strong inhomogeneous electric field and are spatially dispersed according to their effective dipole moments. The red and blue lines show the molecular beam profiles for indole and indole(H_2O), respectively, when the deflector is switched on. These profiles were corrected for the dissociative ionisation of indole$(H_2O)^+$ leading to indole$^+$ [3, 5]. Here, the lower edge of the indole profile is shifted by ∼1 mm whereas for indole(H_2O) it is shifted by ∼1.8 mm, reflecting their different dipole moments of 1.96 D and 4.4 D, respectively [6], with respect to their masses. The blue arrow indicates the position used in the dynamics measurements in this work, where we had an indole(H_2O) sample with an estimated purity of >90%.

Using higher energy laser pulses, we also measured vertical molecular beam profiles for H_2O ($E_i = 12.621$ eV [7]) and $(H_2O)_2$ ($E_i \leq 11.21$ eV [7]), which have a significantly higher ionisation energy E_i than indole(H_2O) ($E_i = 7.37$ eV [8]) and indole ($E_i = 7.76$ eV [7]). The indole(H_2O) signal for this higher laser power, corrected for dissociative ionisation (*vide supra*), is shown in blue in Supplementary Figure 1 b. For improved visibility, the H_2O^+ (green) and $(H_2O)_2^+$ (purple) signals are multiplied by factors of 7 and 22, respectively. For the $(H_2O)_2$ signal, we only took low-kinetic-energy ions into account, which result from single ionisation of $(H_2O)_2$. As can be seen from Supplementary Figure 1 b, $(H_2O)_2$ deflects even further than indole(H_2O), due to the higher dipole-moment-to-mass ratio [9]. The H_2O^+ signal contains contributions from H_2O, $(H_2O)_2$, and indole(H_2O), and therefore extends as far as the $(H_2O)_2$ signal.

SUPPLEMENTARY NOTE 2: DISENTANGLING THE LOW-KINETIC-ENERGY H_2O^+ SIGNAL

Supplementary Figure 1 c shows a VMI image for H_2O^+ recorded in the pure-$(H_2O)_2$ part of the beam. Low-kinetic-energy ions as well as an anisotropic ring resulting from Coulomb explosion of $(H_2O)_2$ into two H_2O^+ were observed. Supplementary Figure 1 d shows a VMI image recorded at the molecular-beam position where the dynamics measurements presented in the main text were performed, i. e., the position marked by the blue arrow in Supplementary Figure 1 a. Here, two additional rings for H_2O^+ were observed due to the Coulomb explosion of indole(H_2O). The Coulomb explosion of indole(H_2O) leading to indole$^+$ and H_2O^+ results in a ring that partly

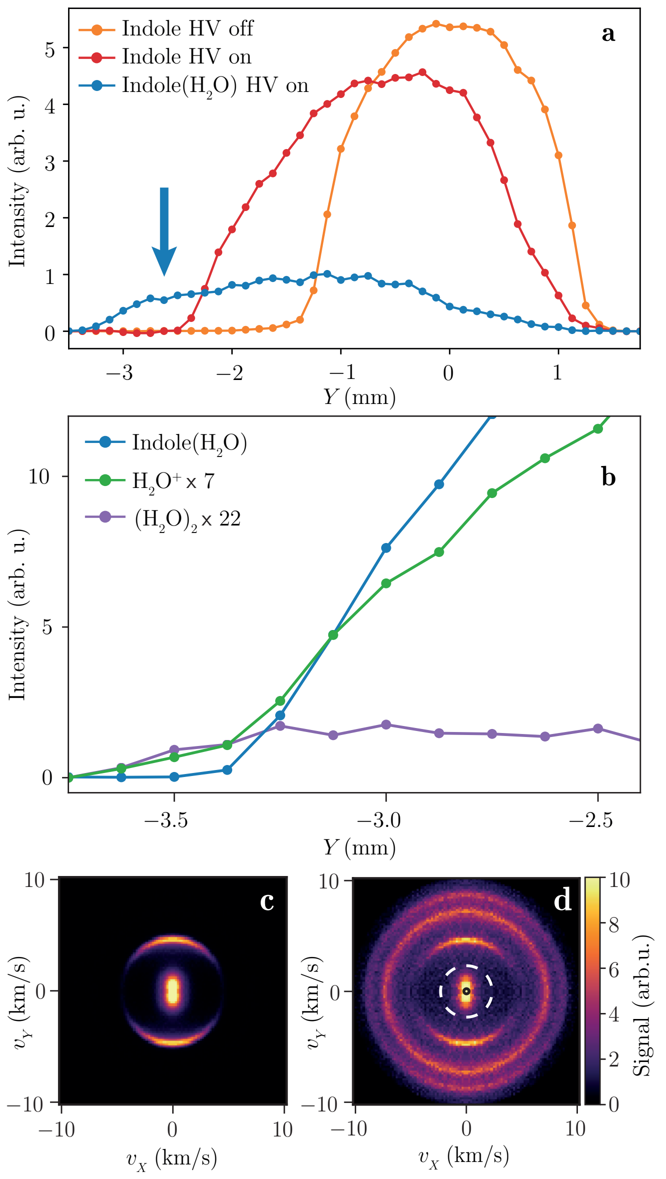

Supplementary Figure 1 | Electrostatic beam deflection and separation of indole(H_2O). **a** Vertical molecular beam column density profiles for indole with the deflector switched off (orange) and on (red), and for indole(H_2O) with the deflector switched on (blue). The blue arrow indicates the position where the dynamics measurements in this work were performed. **b** Vertical molecular beam profiles for indole(H_2O) (blue), H_2O^+ (green), and $(H_2O)_2$ (purple), measured with a higher ionisation-laser power. **c** VMI image for H_2O^+ for a part of the molecular beam with pure $(H_2O)_2$. **d** VMI image for H_2O^+ for the position in the molecular beam where the dynamics measurements were performed. The region between the white dashed circle and the small black circle in the centre indicates the low-kinetic-energy range analysed in the pump-probe experiments. The full images were used for the vertical molecular beam profiles.

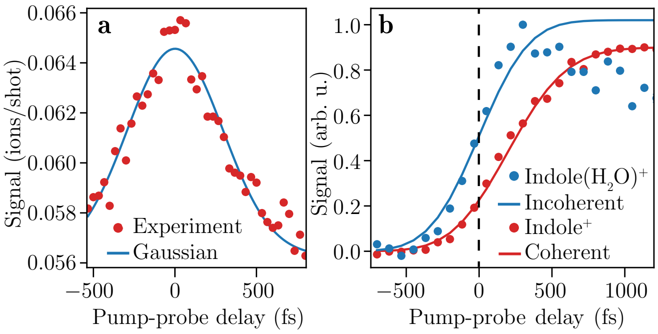

Supplementary Figure 2 | Two measurements of the instrument response function. a, Independent measurement of H_2O^+ signal coming from single ionisation of H_2O. The experimental data points are represented by the red dots and the blue line is a Gaussian fitted to these points, with $\tau_{IRF} = 416$ fs. **b,** High-resolution measurement of the fast increase in the indole$(H_2O)^+$ (blue) and indole$^+$ (red) signals. The dots represent the experimental data points, whereas the blue and red lines represent the incoherent and coherent limits that are fitted to the indole$(H_2O)^+$ and indole$^+$ signals, respectively. This results in an IRF with $\tau_{IRF} = 381$ fs.

overlaps with the ring shown in Supplementary Figure 1 c. Within the dashed white circle in Supplementary Figure 1 d, H_2O^+ signals originating from indole(H_2O) and $(H_2O)_2$ overlap. The region between this circle and the black circle in the centre of the image indicates the low-kinetic-energy ions resulting from neutral dissociation that are used in this paper. We checked that the delay-dependent signal shown by the green squares in Supplementary Figure 1 disappeared when we moved to the pure $(H_2O)_2$ part of the molecular beam. Therefore, we concluded that this delay-dependent signal is due to the neutral dissociation of indole(H_2O).

SUPPLEMENTARY NOTE 3: INSTRUMENT RESPONSE FUNCTION

To determine the temporal overlap of UV and NIR pulses, i. e., t_0, and the temporal instrument response function (IRF) we performed two calibration experiments. Supplementary Figure 2 a shows the H_2O^+ signal as a function of the pump-probe delay in the centre of the molecular beam profile, $Y = 0$ mm, and the centre of the VMI, KE < 120 meV, which corresponds to H_2O^+ signal from single ionisation of H_2O. Fitting a Gaussian to this cross-correlation of the UV and NIR pulses yields an IRF of $\tau_{IRF} = 416$ fs. Assuming that this H_2O^+ signal is due to single ionisation of H_2O using 1 UV and 9 NIR photons [10] and with an NIR probe-pulse duration of 70 fs (FWHM of the intensity envelope, FWHM_I) from an autocorrelation measurement, a UV pulse duration of 692 fs (FWHM_I) is extracted. The mean of the fitted Gaussian yields t_0.

These results were confirmed through a recording of the steep increases in the indole$(H_2O)^+$ and indole$^+$ signals with high temporal resolution. The IRF can be determined from the incoherent and coherent limits of the fast increases [11]. For the incoherent limit, which in our experiment is the case for indole(H_2O) with a dephasing time of 20 . . . 100 fs [12], much smaller than the UV pulse duration, the signal is given by $I_{\text{incoherent}} \propto 1+\text{erf}(t/\tau_{IRF})$ [11]. The increase in the indole$^+$ signal, however, mostly comes from the $\pi\sigma^*$ state, which has a large dephasing time. This allows us to approximate this increase by the coherent-limit formulation $I_{\text{coherent}} \propto [1 + \text{erf}(t/\tau_{IRF})]^2$ [11]. A joint fit of $I_{\text{incoherent}}$ to the indole$(H_2O)^+$ signal and of I_{coherent} to the indole$^+$ signal, Supplementary Figure 2 b, resulted in $\tau_{IRF} = 381$ fs, which is in good agreement with the independent measurement of the IRF above. Thus, this value of $\tau_{IRF} = 381$ fs was used in the evaluation of the Maxwell-Bloch equations.

SUPPLEMENTARY NOTE 4: CONTRIBUTIONS OF BLOCH STATES TO THE ION YIELDS

Supplementary Figure 3 shows the delay-dependent yields of indole$(H_2O)^+$ (left) and indole$^+$ (right) for the long (a, b) and short (c, d) delays. The experimental data is represented by the red dots with vertical lines for the statistical 1σ error estimates. The blue lines show the simulated data based on our reaction model, including the cosine functions to describe the oscillations, see (3) in the main manuscript. From the simulated data, we obtained the contributions of the populations in the different Bloch states ρ_{ii} to the ion signals, which are shown in Supplementary Figure 3 as well. The arbitrarily scaled amplitude of the IRF is shown by the dashed line. The H_2O^+ signal is not shown here, since it only contains the population of the fifth state, ρ_{55}, which is shown in Figure 1 of the main manuscript. We note that the oscillations were undersampled in the long experimental scans, Supplementary Figure 3 a, b, with an experimental stepsize of 1.668 ps similar to the oscillation period. Nevertheless, the fine scans, Supplementary Figure 3 c, d and Supplementary Figure 4, clearly demonstrate the quality of the fit.

States 2 (green) and 3 (orange), corresponding to the $\pi\pi^*$ and $\pi\sigma^*$ states of indole(H_2O), respectively, contribute to the fast increases in the indole$^+$ and indole$(H_2O)^+$ signals. The delayed increase of the indole$^+$ with respect to the indole$(H_2O)^+$ signal is due to the small contribution of state 2 to the indole signal. When the population in state 2 decreases we observe a fast decrease in the indole$(H_2O)^+$ signal. These observations led us to conclude that the fragmentation probability after ionisation from the $\pi\sigma^*$ state, state 3, is larger than from the $\pi\pi^*$ state, state 2. We ascribe this to a higher-energy cationic state that needs to be reached from the $\pi\sigma^*$ state, which is most likely energetically above the dissociation energy of the cation, as described in the main manuscript. In that case, four probe photons are needed to reach this

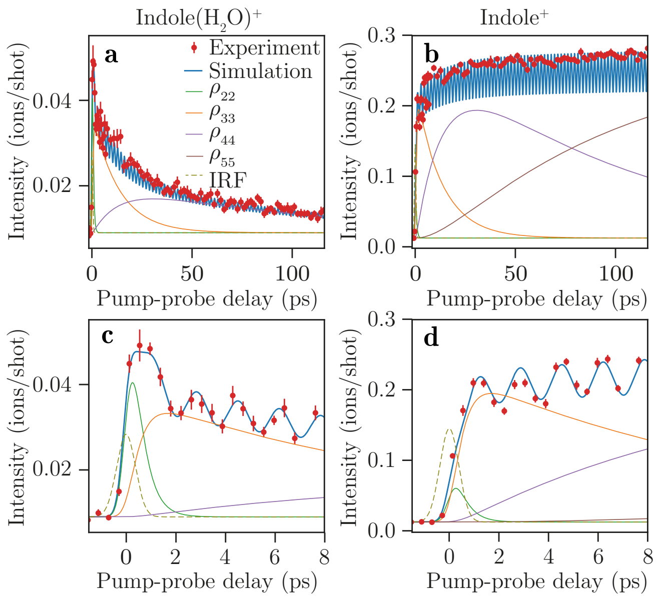

Supplementary Figure 3 | Populations of different Bloch states to the indole(H$_2$O)$^+$ and indole$^+$ signals. The individual contributions of the populations ρ_{ii} in states i to the indole(H$_2$O)$^+$ (left) and indole$^+$ (right) signals for long (**a, b**) and short (**c, d**) pump-probe delays. Statistical 1σ error estimates are given by vertical lines for all experimental data points (red dots). The temporal profile of the IRF (dashed line) is shown on an arbitrary vertical scale. The blue lines show the fits to the experimental data, where the linear combinations of the populations are multiplied by the cosine function to describe the oscillations, see (3) in the main manuscript. In (**a, b**) the oscillations are less pronounced in the experimental data as they are undersampled in these traces, see (**c, d**) and Supplementary Figure 4 for high-resolution data confirming the quality of the fit.

cationic state, instead of three for ionisation from the $\pi\pi^*$ state. A similar effect was observed for indole(NH$_3$): For a probe wavelength of 395 nm the $\pi\pi^*$ state was mostly ionised by a single photon, whereas two photons were needed for ionising the $\pi\sigma^*$ state [13].

The slow decay of the population in state 3 results in a slow decrease in the indole(H$_2$O)$^+$ signal. Due to the contribution of state 4, i. e., the S_0 state of indole(H$_2$O), however, there is still signal left at the longest delays we measured. We assumed that state 5 has no contribution to the indole(H$_2$O)$^+$ signal, since this state corresponds to dissociation into separate indole and H$_2$O molecules. For indole$^+$ the contributions of states 4 and 5 result in an overall slow increase in the signal.

SUPPLEMENTARY NOTE 5: OSCILLATIONS DUE TO WAVEPACKET DYNAMICS

We performed an additional high-temporal-resolution measurement for the indole$^+$ signal to investigate the oscillatory structures we observed, as shown in Figure

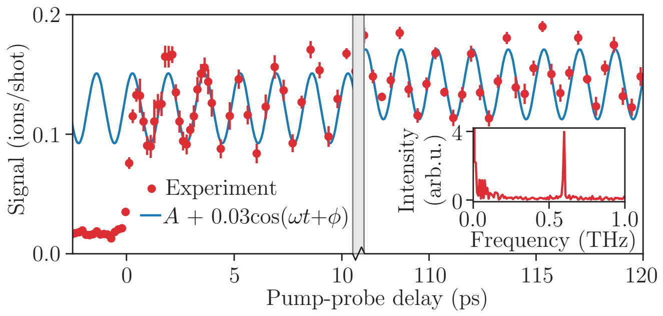

Supplementary Figure 4 | Wavepacket dynamics in indole(H$_2$O). The experimental delay-dependent indole$^+$ signal (red) clearly shows the oscillations. Statistical 1σ error estimates are given by vertical lines for all data points. The blue line represents a cosine function and the inset shows a Fourier transform of the indole$^+$ signal, see the text for more details.

1 b of the main manuscript. In these measurements, we used a step size of 167 fs for $t = -2.544\ldots3.962$ ps and increased the step size to 417 fs for longer delays up to 120 ps. The oscillations are clearly present for all delays. In Supplementary Figure 4, red dots with vertical lines for the statistical 1σ error estimates show the experimental results for $t = -2.5\ldots10.5$ ps and $t = 107\ldots120$ ps. The inset shows a Fourier transform of the indole$^+$ signal for delays between 3.962 ps and 120 ps, where the sampling frequency is constant. There is one distinct peak visible with a frequency of $f = 0.60$ THz, which corresponds to 20 cm^{-1} or a period of $T = 1.67$ ps.

In order to examine the damping of the oscillations, we compared the experiment with the function $y(t) = A + B\cos(\omega t + \phi)$. We fitted this function to our data points for t<10 ps and found that $A = 0.12, B = 0.03, \phi = -1.0$ and $\omega = 2\pi \cdot 0.60$ THz, corresponding to 20 cm^{-1} and $T = 1.67$ ps. This function is shown by the blue line in Supplementary Figure 4 for short delays. Due to the overall slow increase in the indole$^+$ signal for longer delays, A depends on the pump-probe delay. To account for this, we show the same function but with $A = 0.14$ for the long delays in Supplementary Figure 4. This clearly shows that the oscillations are hardly damped, if at all, on a timescale of 120 ps. We also observed these oscillations with a period of $T = 1.67$ ps in the indole$^+$ signal coming from bare indole (not shown).

Oscillations with a frequency of 23 ± 5 cm^{-1} ($T = 1.45$ ps) were previously described for indole(NH$_3$) [14, p. 131–133]. These were found to not depend on the ammonia isotopologue, IndND(ND$_3$) or indole(NH$_3$), nor on pump wavelength. These oscillations were interpreted in terms of coherent wavepacket dynamics, where the frequency suggests that low-frequency, presumably intermolecular, vibrational modes are involved [14, p. 131–133].

However, as these oscillation frequencies are similar for indole, indole(H$_2$O), and indole(NH$_3$) and as the oscillations are hardly damped over >100 ps, we assign them to coherent wavepackets of vibrational modes in indole

that are not directly involved in the dissociation process, but modulate the ionisation probability for our probe step. In bare indole, there are two in-plane modes with observed vibrational frequencies of 1489 and 1510 cm^{-1} and several out-of-plane modes, e. g., with observed frequencies of 715, 738, and 762 cm^{-1}, which all exhibit an energy spacing of $\sim$20 cm^{-1} [15] and are, therefore, consistent with our observations. Other possible candidates for these dynamics would be the C–H stretching vibrations with fundamental excitation wavenumbers of 3031, 3054, 3070, and 3091 cm^{-1} for indole(H_2O) [16] and at 3051, 3072, and 3090 cm^{-1} for indole [17] that all exhibit energy spacings of $\sim$20 cm^{-1}.

The bandwidth of our UV pulses of 4.5 nm (FWHM), see "Methods", corresponding to 565 cm^{-1}, allowed to excite wavepackets over many of these states. The effective NIR bandwidth of $\sim$850 cm^{-1} is sufficient to probe the complete energy range in a 3-photon ionisation process.

SUPPLEMENTARY REFERENCES

[1] S. Trippel, Y.-P. Chang, S. Stern, T. Mullins, L. Holmegaard, and J. Küpper, Spatial separation of state- and size-selected neutral clusters, Phys. Rev. A **86**, 033202 (2012), arXiv:1208.4935 [physics].

[2] Y.-P. Chang, D. A. Horke, S. Trippel, and J. Küpper, Spatially-controlled complex molecules and their applications, Int. Rev. Phys. Chem. **34**, 557 (2015), arXiv:1505.05632 [physics].

[3] S. Trippel, M. Johny, T. Kierspel, J. Onvlee, H. Bieker, H. Ye, T. Mullins, L. Gumprecht, K. Długołęcki, and J. Küpper, Knife edge skimming for improved separation of molecular species by the deflector, Rev. Sci. Instrum. **89**, 096110 (2018), arXiv:1802.04053 [physics].

[4] S. Trippel, J. Wiese, T. Mullins, and J. Küpper, Communication: Strong laser alignment of solvent-solute aggregates in the gas-phase, J. Chem. Phys. **148**, 101103 (2018), arXiv:1801.08789 [physics].

[5] M. Johny, J. Onvlee, T. Kierspel, H. Bieker, S. Trippel, and J. Küpper, Spatial separation of pyrrole and pyrrole-water clusters, Chem. Phys. Lett. **721**, 149–152 (2019), arXiv:1901.05267 [physics].

[6] C. Kang, T. M. Korter, and D. W. Pratt, Experimental measurement of the induced dipole moment of an isolated molecule in its ground and electronically excited states: Indole and indole–H_2O, J. Chem. Phys. **122**, 174301 (2005).

[7] P. J. Linstrom and W. G. Mallard, eds., *NIST Chemistry WebBook, NIST Standard Reference Database Number 69* (National Institute of Standards and Technology, Gaithersburg MD, 20899, 2017).

[8] J. E. Braun, T. L. Grebner, and H. J. Neusser, Van der Waals versus hydrogen-bonding in complexes of indole with argon, water, and benzene by mass-analyzed pulsed field threshold ionization, J. Phys. Chem. A **102**, 3273 (1998).

[9] H. Bieker, J. Onvlee, M. Johny, L. He, T. Kierspel, S. Trippel, D. A. Horke, and J. Küpper, Pure molecular beam of water dimer, J. Phys. Chem. A **123**, 7486 (2019), arXiv:1904.08716 [physics].

[10] D. Yeager, V. McKoy, and G. A. Segal, Assignments in the electronic spectrum of water, J. Chem. Phys. **61**, 755 (1974).

[11] I. V. Hertel and W. Radloff, Ultrafast dynamics in isolated molecules and molecular clusters, Rep. Prog. Phys. **69**, 1897 (2006).

[12] A. Peralta Conde, V. Ovejas, R. Montero, F. Castaño, and A. Longarte, Influence of solvation on the indole photophysics: Ultrafast dynamics of indole–water clusters, Chem. Phys. Lett. **530**, 25 (2012).

[13] H. Lippert, V. Stert, L. Hesse, C. P. Schulz, I. V. Hertel, and W. Radloff, Analysis of Hydrogen Atom Transfer in Photoexcited Indole$(NH_3)_n$ Clusters by Femtosecond Time-Resolved Photoelectron Spectroscopy, J. Phys. Chem. A **107**, 8239 (2003).

[14] H. Lippert, *Ultrakurzzeitspektroskopie von isolierten und mikrosolvatisierten Biochromophoren*, Ph. D. thesis, Freien Universität Berlin, Berlin, Germany (2004).

[15] C. Brand, J. Küpper, D. W. Pratt, W. Leo Meerts, D. Krügler, J. Tatchen, and M. Schmitt, Vibronic coupling in indole: I. theoretical description of the ^1L_a-^1L_b interaction and the electronic spectrum, Phys. Chem. Chem. Phys. **12**, 4968 (2010).

[16] C. Unterberg, A. Jansen, and M. Gerhards, Ultraviolet/infrared-double resonance spectroscopy and *ab initio* calculations on the indole$^+$ and indole$(H_2O)_1^+$ cations, J. Chem. Phys. **113**, 7945 (2000).

[17] B. J. Smith and R. Liu, A theoretical investigation of indole tautomers, J. Mol. Struct. – Theochem **491**, 211 (1999).
